# Colonization profiles of gut microbiota in goat kids from neonatal to weaning period

**DOI:** 10.3389/fmicb.2024.1467205

**Published:** 2024-10-01

**Authors:** Yuqin Wu, Dunying Hou, Siyuan Zhan, Linjie Wang, Jiaxue Cao, Jiazhong Guo, Li Li, Hongping Zhang, Lili Niu, Tao Zhong

**Affiliations:** Farm Animal Genetic Resources Exploration and Innovation Key Laboratory of Sichuan Province, College of Animal Science and Technology, Sichuan Agricultural University, Chengdu, China

**Keywords:** bacterial colonization, diet, fecal microbiota, goat, weaning

## Abstract

Understanding the colonization and change patterns of gut microbiota is pivotal for comprehending host health. As a newly cultured breed, the studies on the gut microbiota of Tianfu goats remain limited. This study aimed to address this gap by analyzing the microbial composition and colonization patterns of fecal samples collected from goat kids from birth to weaning. Fecal samples were collected on days 0, 7, 14, 21, 28, 35, 42, 49, 53, 55, 57, and 64, and the changes and colonization patterns of microorganisms were analyzed through high-throughput 16S rRNA sequencing. The results showed that the abundance of fecal microbiota in goat kids gradually increased over time, followed by a decrease after weaning and stabilization, with reduced individual differences. The colonization of fecal microorganisms mainly presented three different stages: days 0–14, days 21–49, and days 53–64. During the suckling period, the relative abundance of *Proteobacteria* (72.34%) was the highest, followed by *Firmicutes* (21.66%). From 21 days old, the microbiota in goat kids gradually to be diverse, with *Lachnospiraceae* and *Ruminococcaceae* being dominant. During post-weaning, *Ruminococcaceae* (30.98–33.34%) was becoming prominence which helpful for cellulose decomposition. LEfSe analyzed three important time points (d0 vs. d7, d7 vs. d14, d49 vs. d53, LDA score > 4 and *p* < 0.05), 53 microbial communities with stage differences were identified. Functional prediction using PICRUSt revealed that differential microbial communities are mainly related to carbohydrate and amino acid metabolism pathways. Overall, this study addresses the intricate relationship between ages, diets, and microbiota compositions in Tianfu goat kids, and also offering insights into microorganisms-host interactions.

## 1 Introduction

The gastrointestinal tract (GIT) of mammals harbors a vast array of microbial inhabitants, which play pivotal roles in host development, physiology, and health (Lynch and Pedersen, 2016). As one of the important sites for animal digestion and absorption of nutrients, many microbiota are also colonized in the GIT. These microbiotas mainly rely on animal intestines for survival, while assisting the host in completing various physiological and biochemical functions, ultimately forming a stable microecological balance with the host (Sonnenburg et al., 2004). The composition of gut microbiota is intricately influenced by the host’s specific gut environment, age, medicine, and other factors. Meanwhile, the diversity of animal gut microbiota often undergoes some regular changes with the increase of animal age (Jäger et al., 2019).

Importantly, microbial colonization in the GIT of animals after birth has long-term effects on host development, as well as adult health and phenotype (Fan et al., 2021; Furman et al., 2020). In juvenile ruminants, the composition of gut microbiota is primarily influenced by age and diet (Guo et al., 2020; Maslowski and Mackay, 2011). In the study of rumen development, it was found that it can be divided into three stages: non ruminant stage (0–21 days), transitional stage (21–56 days), and ruminant stage (from day 56 onwards), and the corresponding changes in microbial relative abundance also showed phased changes (Ferretti et al., 2018; Rey et al., 2014; Wardrop and Coombe, 1960). The diversity of microbial communities in the intestine also exhibited similar patterns. The diversity of gut microbiota gradually increased from birth to 2.5 years old in newborns (Koenig et al., 2011). Guo et al. (2020) analyzed the gut microbiota of 0–56 day old goat kids and found that as age increased, the richness of the microbiota increased, and the relative content of the microbiota tended to stabilize. Similar studies have also found in human infants that the diversity and similarity of gut microbiota in six infants increase with age (Palmer et al., 2007). The specific colonization and change patterns of microbiota in the gut of goat kids from birth to weaning, as well as the dominant microbiota at each stage, are not yet clear and require further experimental analysis.

The gut microbiota plays a crucial role in regulating the host’s metabolism, physiology, and immune response (Nicholson et al., 2012; Sonnenburg and Ang Bäckhed, 2016). When the relatively stable gut microbiota experiences disruptions, it can lead to microbial ecological imbalances, potentially resulting in intestinal ailments such as ulcerative colitis and Crohn’s disease (Kuhn et al., 2014; Nishida et al., 2018). Appropriately supplementing probiotics can help boost the population of beneficial bacteria in the intestine while simultaneously reducing levels of harmful bacteria (Heak et al., 2018). Wu et al. (2021) found that the gut microbiota has a positive impact on lipid metabolism by inhibiting the activity of lipoprotein lipase in adipocytes. The function of the gut microbiota was closely related to its diet during the growth process of young ruminants. During the transition from colostrum to regular milk, there is a gradual increase in the proportion of bacteria capable of utilizing the nutrients present in milk (Dias et al., 2017; Ferretti et al., 2018). When the proportion of breast milk intake decreased, the intake of solid feed and crude fiber gradually increased, and the proportion of microorganisms that can degrade starch and fiber gradually increased, becoming the dominant bacterial species (Kim et al., 2019). Under different dietary conditions, the composition of gut microbiota in young ruminants varies, which helps them better adapt to environmental changes.

As a significant goat-producing nation, our country has a wide variety of local goat breeds, but there are problems such as low level of breeding and poor production performance of some breeds. Tianfu goat is a new breed obtained by crossbreeding Chengdu Grey goats with Saanen, Toggenburg, Nubian and Boer goats (Wang et al., 2011). It has wide adaptability, strong disease resistance, tolerance to rough feeding, and excellent meat performance, especially suitable for large-scale house feeding. At present, there is no research on the changes in fecal microbiota of Tianfu goat kids from birth to weaning, and there is a lack of comprehensive understanding of the acquisition and establishment of early life gut microbiota in goat kid. Understanding the characteristics of healthy gut microbiota in normal individuals can help better address intestinal diseases caused by microbial imbalance. In the current study, we collected fecal samples from 20 healthy Tianfu goat kids at 12 time points from birth to weaning to investigate the establishment and changes of gut microbiota in healthy goat kid, and provided a framework for designing better strategies to intervene in gut microbiota to improve health or products.

## 2 Materials and methods

### 2.1 Animal management and sample collection

Tianfu goats were raised in the Goat Breeding Farm of Sichuan Agricultural University (Yaan, Sichuan, China, 29°58’ N and 102°59′ E). In order to systematically study the changes and colonization patterns of fecal microbiota in goat kids from birth to weaning, 15 ewes were induced to concentrate estrus during a specific time period through estrus synchronization, and generated 29 goat kids were born in total. All the newborn goat kids were raised in a same environment. Ten health male kids (birthweight, 3.70 ± 0.77 kg) and ten female kids (3.29 ± 0.56 kg) were selected to investigate the colonization pattern of gut microbiota. All kids were housed by lactating ewes from d1 to d49 in goatcotes in groups of 5 ewes and their kids (approximate 2 m^2^ per animal). The goat kids were supplemented the supplementary feed for goat kids on d8 (Table 1). Kids were weaned and separated from ewes on d50. It was worth noting that five days prior to weaning, goat kids were subjected to pre-weaning adaptability training. A single batch of goat kids was driven to separate pens away from the mother pen for feeding at 8:00 h and returned to the pen at 18:00 h. During this period, it was ensured that there was no communication of vocalizations, behaviors, and odors between the ewe and the goat kid. During the trial, no antibiotics were added to their diet and no drugs were injected. All goat kids had been vaccinated with relevant vaccines (ovine braxy, struck, lamb dysentery, enterotoxemia).

**Table 1:** Ingredients and chemical composition of supplementary feed for goat kids (dry-matter basis).

Items	Content
Ingredient (%)	
Corn	50.00
Soybean meal	18.00
Corn germ meal	15.00
Grass powder	6.50
Bran	6.00
Calcium carbonate	1.5
NaCl	1.00
NaHCO_3_	1.00
Premix^1^	1.00
Nutritional level (%)	
CP	14.61
EE	2.30
Ash	11.13
NDF	42.39
ADF	20.96
Ga	0.68
P	0.18

^1Each 1 kg premix contained 3,500 IU vitamin A, 1,000 IU vitamin D, 40 IU vitamin E, 50 mg iron, 40 mg manganese, 10 mg copper, 40 mg Zinc, 0.3 mg selenium. CP, crude protein; EE, crude fat; Ash, crude ash content; NDF, neutral detergent fiber; ADF, acidic detergent fiber.^

Fresh feces were collected from 20 experimental goat kids at 12 time points (0, 7, 14, 21, 28, 35, 42, 49, 53, 55, 57, 64 days (d) of age). Fecal collection began after the feed was placed at 8:00 h each day. Notably, the feces of goat kids on d0 of birth were collected within 24 h after the kids had eaten their breast milk after birth. The sterile swab was inserted about 1–1.5 cm into the anus to stimulate defecation, and stool was collected in a cryopreservation tube and immediately placed into −80 for frozen storage. From July to September 2023, a total of 240 fecal samples were collected. All the sampling procedures were reviewed and approved by the Animal Care and Use Committee of Sichuan Agricultural University (Permit number, Dky-2022202021).

### 2.2 DNA extraction and 16S rRNA sequencing

Total microbial DNA was extracted from the collected samples using the Magnetic Soil and Stool DNA Kit (Tiangen, Beijing, China) according to the manufacturer’s instructions. The extracted DNA was quantified using a NanoDrop (Thermo Fisher Scientific, Wilmington, DE, USA). Amplification of the V3-V4 variable regions within the 16S rRNA gene sequence was achieved through the use of specific primers: 341F (5’-CCTACGGGNGGCWGCAG-3′) and 805R (5’-GACTACHVGGGTATCTAATCC-3′). The conditions for PCR amplification were as follows: initial denaturation for 30 s at 98°C; 32 cycles of 10 s at 98°C, 30 s at 50°C, and 45 s at 72°C; and a final elongation step for 10 min at 72°C. The amplicons were sequenced on the Illumina MiSeq platform (Lianchuan Biotechnology Co., Ltd., Zhejiang, China), generating paired-end reads.

### 2.3 Microbial data analysis

Utilize Overlap to stitch together paired-end data, and perform quality control and chimera filtering to obtain high-quality clean data. The clean data were further processed using the DADA2 method in QIIME2 (v 2023.05) to generate a produce table of amplicon sequence variants (ASVs) (Callahan et al., 2016). To avoid the interference of contingent opportunistic factors and low abundance feature sequence, the table were filtered using the qiime feature-table filter-features commands (−-p-min-frequency 2). This step ensures that only those feature sequences are retained which have a cumulative sequencing read count exceeding 2 reads across all samples. Before proceeding with downstream processing, the data were reduced to the minimum library size (commands: --p-sampling-depth 2,710) to obtain the final ASV count data for six taxonomic levels (from phylum to species). Perform species annotation of samples using Greengenes2 (v 2022.10) (McDonald et al., 2024). Alpha diversity, including the Shannon, Chao 1, ACE, and Richness, were calculated based on the ASV feature table using the “diversity” function in the R (v 4.3.0) package. The principal coordinate analysis (PCoA) was conducted based on the Bray-Curtis distance. The linear discriminant analysis (LDA) effect size (LEfSe) is a tool that can be used to identify feature microbial species that exhibit significant differences across various growth stages (Segata et al., 2011). LDA score > 4 and *p* < 0.05 was used as a criterion for judging the significant microbial effect size (Lin et al., 2023). To elucidate the functional roles executed by the microbial community *in vivo*, we conducted a predictive analysis of 16S rRNA gene sequences using the PICRUSt2 (v 2.5.2) software (Douglas et al., 2020).

### 2.4 Statistical analysis

The differences between groups were evaluated using the Wallace-Duncan test, conducted using SPSS (v 19.0) software. The data were expressed as the mean ± standard error of the mean (SEM) and analyzed to calculate the relationship between groups using GraphPad Prism (v 5.0). *p* < 0.05 was considered statistically significant, *p* < 0.01 was considered extremely significant.

## 3 Results

### 3.1 Fecal microbiota differentiates with the age of goat kids

**Figure 1:**
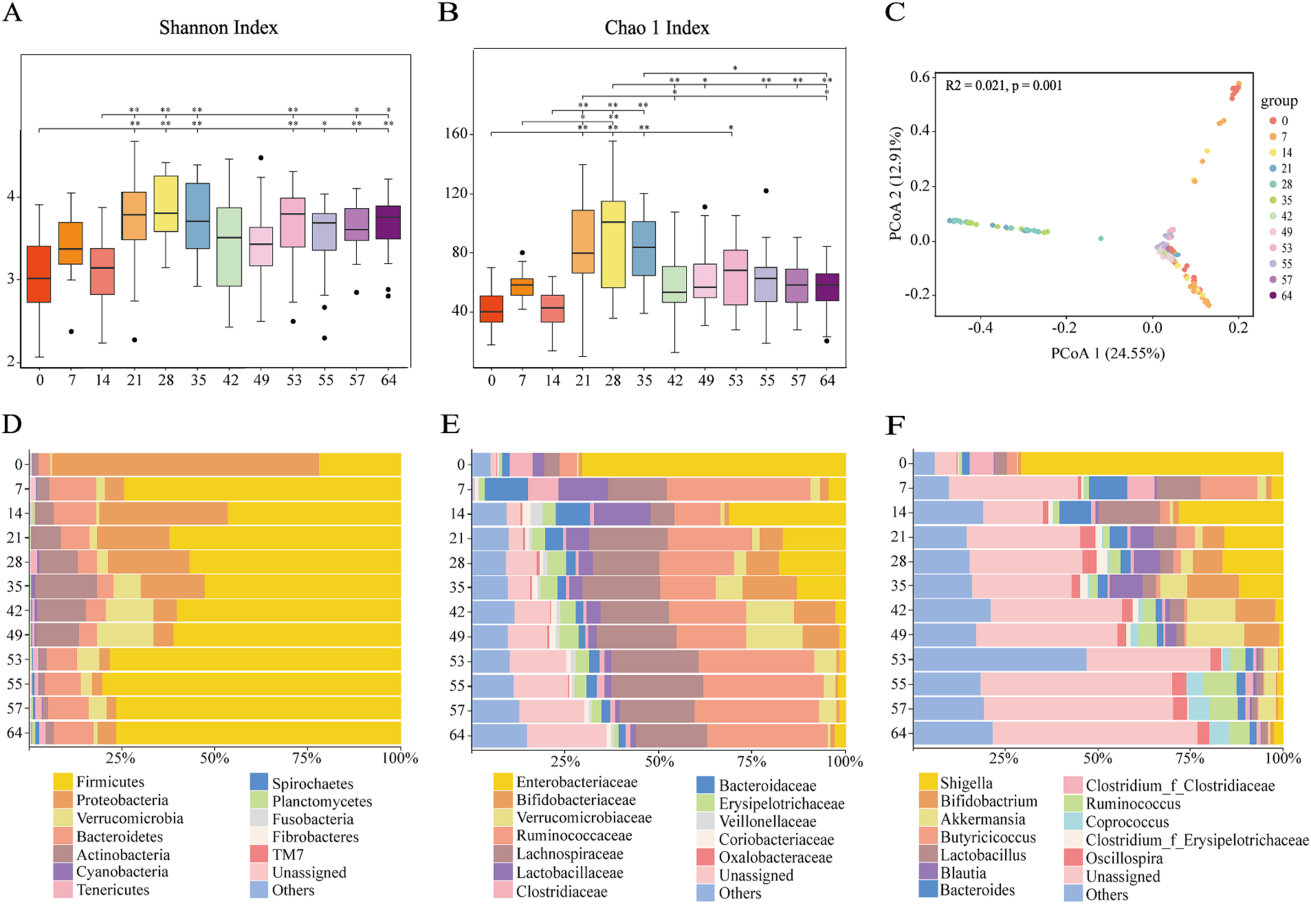
Fecal microbiome changes with ages of goat kids in all the twelve age groups. **(A)** Shannon index. **(B)** Chao 1 index. **(C)** Principal coordinates analysis (PCoA) of all the 240 microbial samples using the Bray-Curtis distance. The bacterial community composition at **(D)** the phylum level, **(E)** the family level, **(F)** the genus level in feces of goat kids in the twelve age groups. The top 12 bacterial are represented separately with different colors, whereas the “Others” category represents other known low abundance bacteria, and “Unassigned” represents bacteria that have not been annotated (*n* = 20, male kid = 10, female kid = 10).

We sequenced the 16S rRNA of 240 stool samples of goat kids from 0, 7, 14, 21, 28, 35, 42, 49, 53, 55, 57, and 64 days old. Detailed sequencing statistics can be found in Supplementary Table S1. The rarefaction curves constructed using both the Shannon Index and Observed richness approach a plateau, indicating that the sampling has sufficient sequence coverage to accurately describe the microbial composition of each group (Supplementary Figure S1). According to the Shannon and Chao 1 Index, the diversity of bacterial communities in goat kid feces significantly increases with age. We observed a significant increase (*p* < 0.01) in the fecal microbiota of 14 to 35 day old goat kids. There was a significant difference (*p* < 0.01) in the Chao 1 Index of fecal microbiota in goat kids from d28 to d64 after weaning (Figures 1A,B). The temporal changes in microbial structure based on the PCoA (R2 = 0.021, *p* = 0.001) (Figure 1C). The cluster of d42 to d64 samples were clustered together, while d0, d7 and d14 were clustered separately compared with the d21 to d35.

### 3.2 Fecal microbial composition and differences

**Figure 2:**
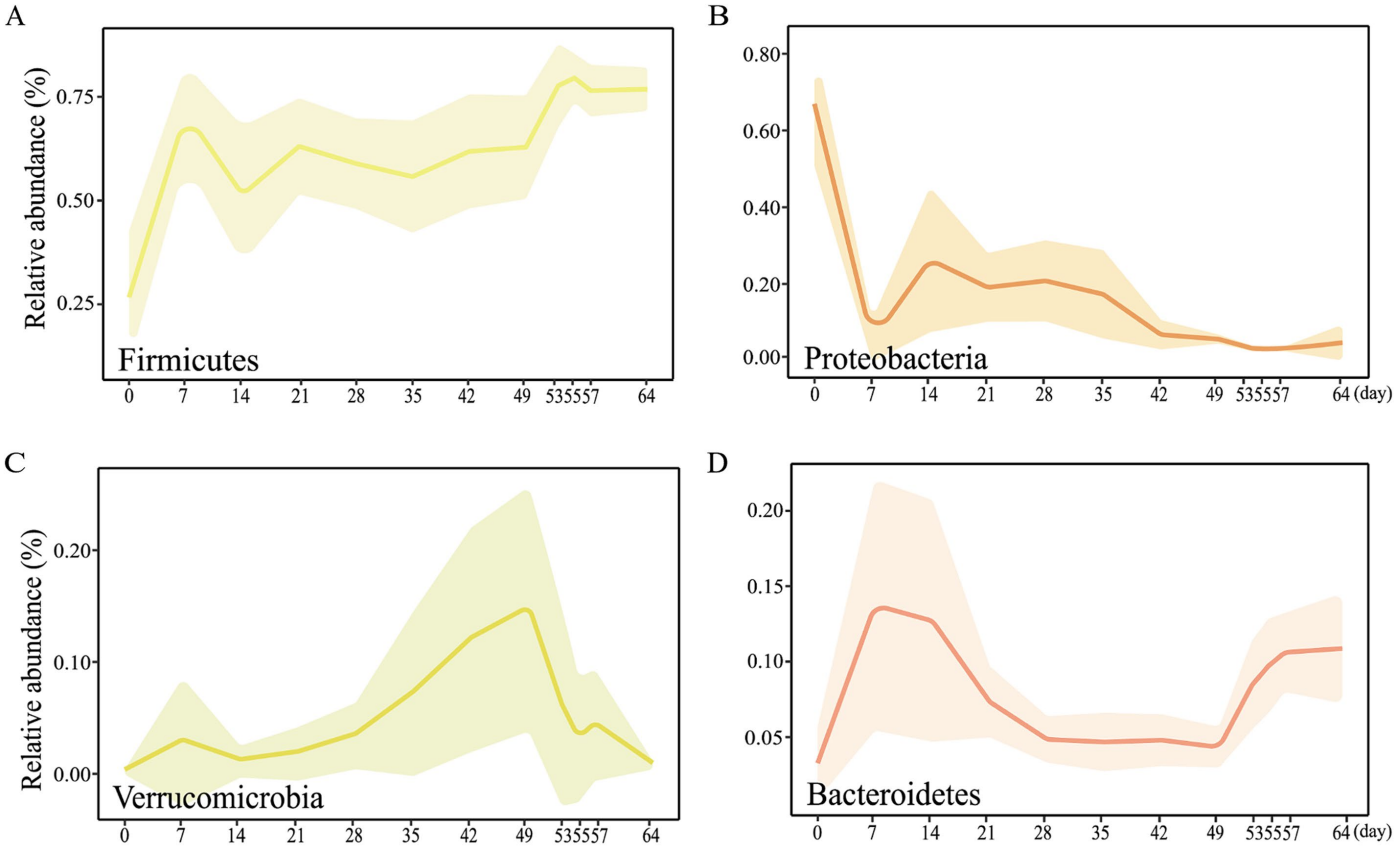
Main bacterial communities at the phylum level from d0 to d64. Dynamic changes of relative abundance of the microbial phyla **(A)** *Firmicutes*, **(B)** *Proteobacteria*, **(C)** *Verrucomicrobia*, **(D)** *Bacteroidetes*.

**Figure 3:**
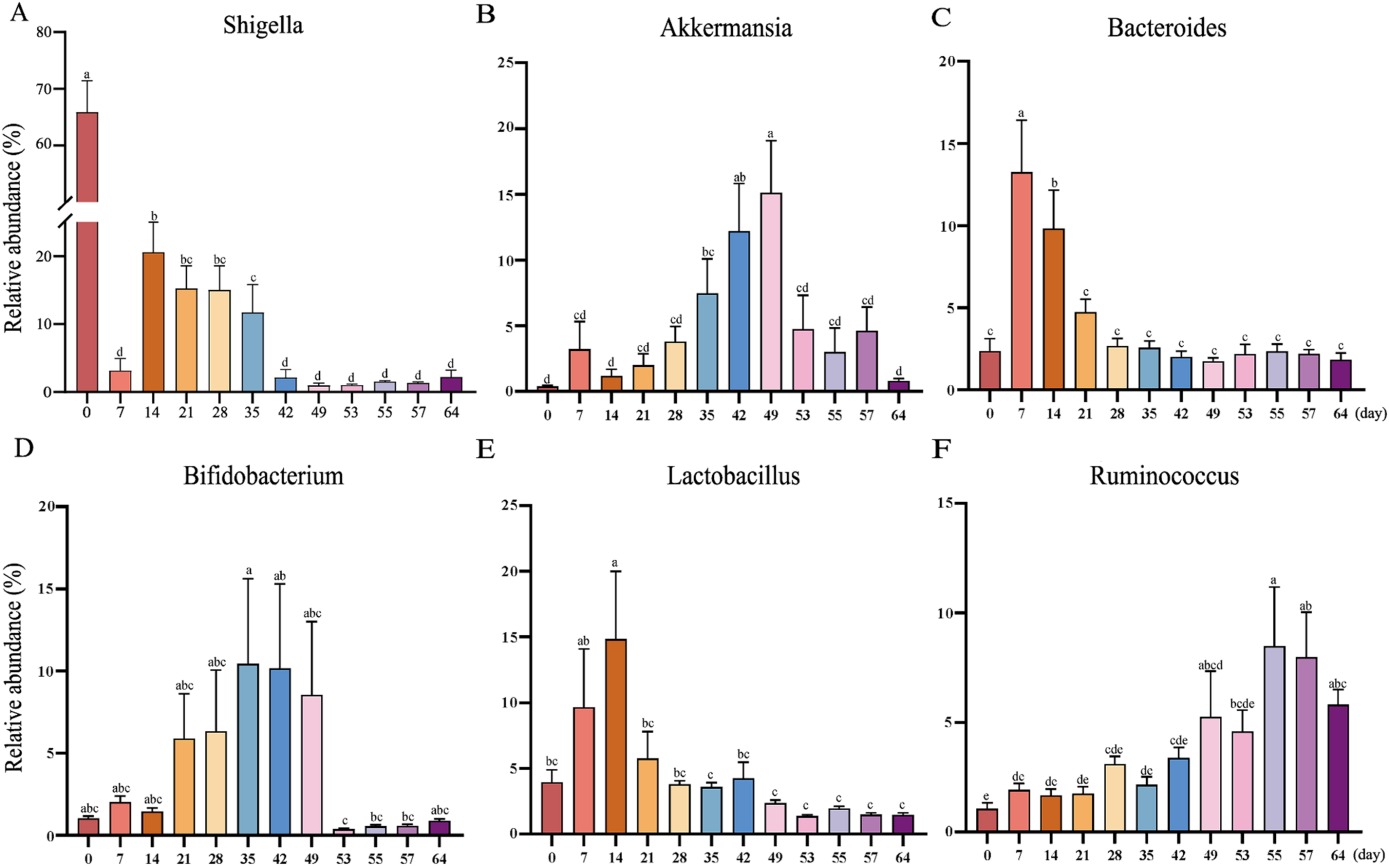
The abundances of the top 6 bacterial genera in goat kid fecal microbiota from d0 to d64. **(A)** *Shigella*, **(B)** *Akkermansia*, **(C)** *Bacteroides*, **(D)** *Bifidobacterium*, **(E)** *Lactobacillus*, **(F)** *Ruminococcus*. Error here represents standard error of means. ^a,b,c,d,e^The same letter indicates no significant difference, whereas different letter indicates significant differences (*p* < 0.05, *n* = 20, male kid = 10, female kid = 10).

At the phylum level, the relative abundance of *Firmicutes*, *Proteobacteria*, *Verrucomicrobia*, and *Bacteroidetes* was extremely high (Figure 1D). Included in these groups, *Ruminococcaceae* and *Lachnospiraceae*, two family members in the *Firmicutes* phylum, were nearly major bacteria across all samples (Figures 1D,E). The relative abundance of *Ruminococcaceae* was lowest on d0, and the highest on d7, and was similar between d14 and d49. After weaning, the relative abundance increased and tended to be stable. The trend of relative abundance of *Ruminococcaceae* is basically consistent with the trend of *Firmicutes* (Figure 2A). *Lachnospiraceae* was among the multiple families with comparable abundance from d0 to d14, but exhibited a rising trend from d21 to d55. *Proteobacteria* and its subfamily *Enterobacteriaceae* were enriched on d0, but the abundance gradually decreased with age (Figures 1E, 2B). According to the experimental results, *Verrucomicrobiaceae* of the phylum *Verrucomicrobia* was found to be almost non-existent in the early stage of the experiment, increasing from d28, and reaching a maximum relative abundance at d49 (15.17%). The relative abundance gradually decreased after weaning (d53), and its trend was similar to that of *Verrucomicrobia* (Figure 2C). The relative abundance of *Bifidobacteriaceae* in *Actinobacteria* phylum, which had similar changes to *Verrucomicrobiaceae*, increased gradually from d21, but decreased significantly after weaning (d53). The relative abundance of *Bacteroidetea* showed a significant increase on the d7, but decreased and gradually stabilized after the d21 (Figure 2D). The changing trend of *Bacteroidaceae* was consistent with it. At the genus level, *Shigella* (*Enterobacteriaceae* family) had the highest relative abundance on d0 (Figure 1F), significantly higher than all other time (Figure 3A), with a relative abundance of 1.57% after weaning (d53 to d64) (Supplementary Table S2). A*kkermansia* of the family *Verrucomicrobiaceae* had the highest abundance on d49 (15.13%), and the lowest relative abundance on d0 and d64 (0.39 and 0.82%) (Figure 3B). *Bacteroides* (*Bacteroidaceae* family) reached its highest relative abundance on d7 (13.26%), significantly higher than other time, and its relative abundance gradually stabilized after d28 (Figure 3C). Throughout the experimental process, *Ruminococcus* of the family *Ruminococcaceae* showed a steady increase in relative abundance, reaching its highest level on the fifth day after weaning (d55) (Figure 3F). Moreover, some microbiota was enriched at certain ages. For example, *Bifidobacterium* (*Bifidobacteriaceae* family) showed a stable increase in relative abundance from d21 to d49, and obviously decreased after weaning (d53 to d64) (Figure 3D). *Lactobacillus* (*Lactobacillaceae* family) was enriched on d14 (14.85%), after which its relative abundance significantly declined, and it tended to stabilize after weaning (d53 to d64) (Figure 3E). The microbial content showed regular changes: the relative abundance of microorganisms fluctuated greatly from d0 to d14; the microbial composition tended to stabilize from d21 to d49; after weaning (d53-d64), the microbial composition and relative abundance changed and quickly reached relative stability.

### 3.3 LEfSe analysis revealed differential microbiota at various time points

**Figure 4:**
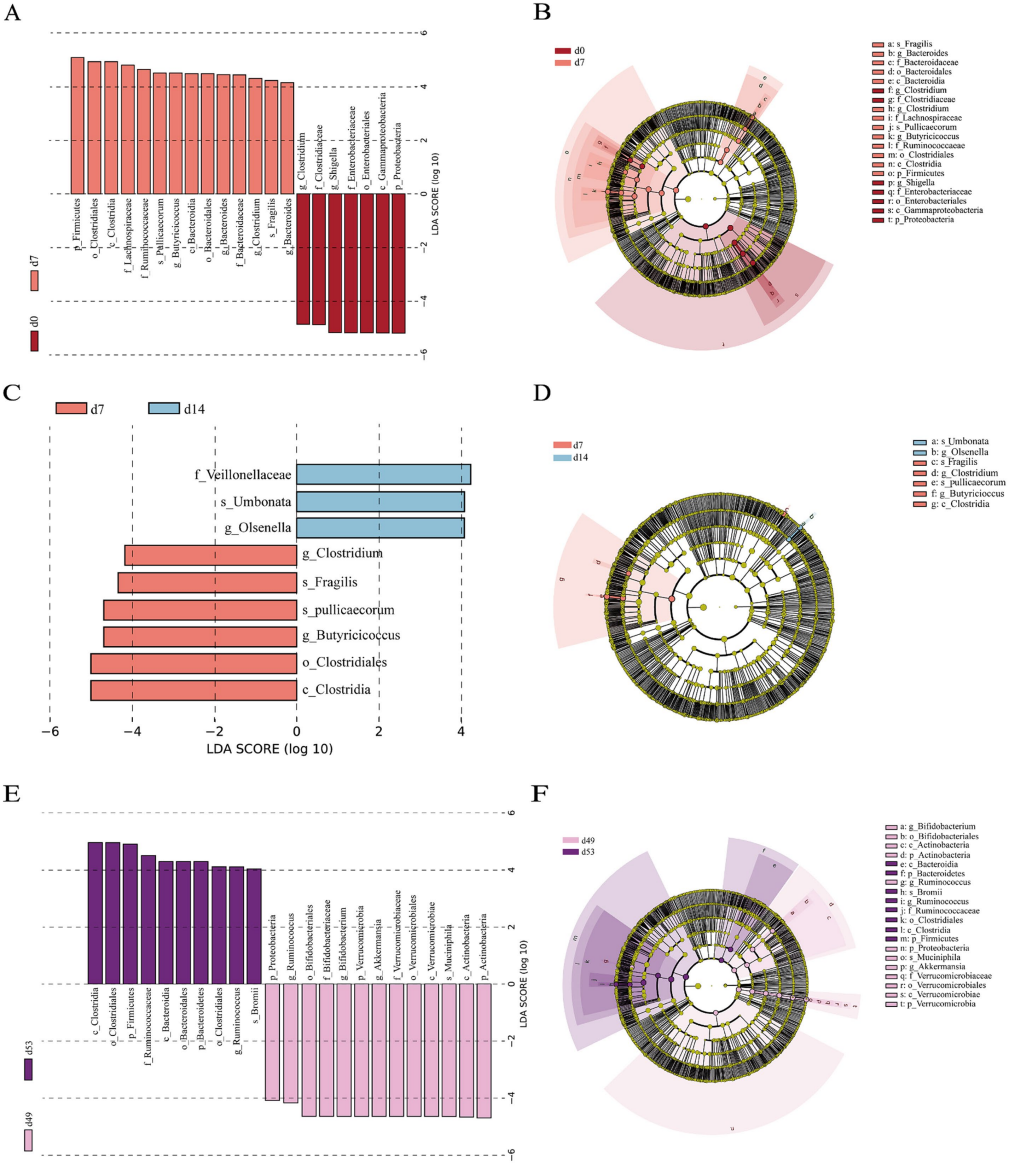
Differential fecal bacterial taxa analyzed by linear discriminant analysis effect size (LEfSe) analysis at different time points. **(A)** Histogram and **(B)** taxonomic cladogram between d0 and d7. **(C)** Histogram and **(D)** taxonomic cladogram between d7 and d14. **(E)** Histogram and **(F)** taxonomic cladogram between d49 and d53. LDA score > 4 and *p* < 0.05, *n* = 20, male kid = 10, female kid =10.

We conducted a differential characterization of bacterial abundance changes in the feces of goat kids at various time points using LEfSe analysis (LDA scores >4 and *p* < 0.05) (Figure 4), where the variations were primarily driven by the influences of diet and age. On the day of birth (d0) when the goat kids were solely consuming colostrum, the abundance of *Shigella* from the *Enterobacteriaceae* family and *Clostridium* from the *Clostridiaceae* family were significantly higher than at d7. Meanwhile, *Pullicaecorum*, *Fragilis*, *Clostridium*, *Bacteroidaceae*, *Lachnospiraceae* and *Butyricicoccus* were enriched in goat kids at d7 (Figures 4A,B). As the goat kids gradually grew and began to consume solid feed, *Umbonata*, *Olsenella* and *Veillonellaceae* were enriched on the d14, whereas the abundance of *Fragilis*, *Pullicaecorum*, *Clostridium*, *Butyricicoccus*, and *Clostridiales* were enriched on the d7 (Figures 4C,D). Moreover, significant changes in the microbial composition of goat kids were observed before and after weaning, *Bromii*, *Ruminococcus*, *Ruminococcaceae*, *Clostridiales*, *Bacteroidales*, and *Bacteroidia* were enriched after weaning (d53), whereas the abundance of *Muciniphila*, *Akkermansia*, *Bifidobacterium*, *Ruminococcus*, *Verrucomicrobiaceae*, *Verrucomicrobiales*, *Bifidobacteriaceae*, and *Bifidobacteriales* were enriched on the d49 (Figures 4D,E).

### 3.4 Microbial function prediction at various time points

**Figure 5:**
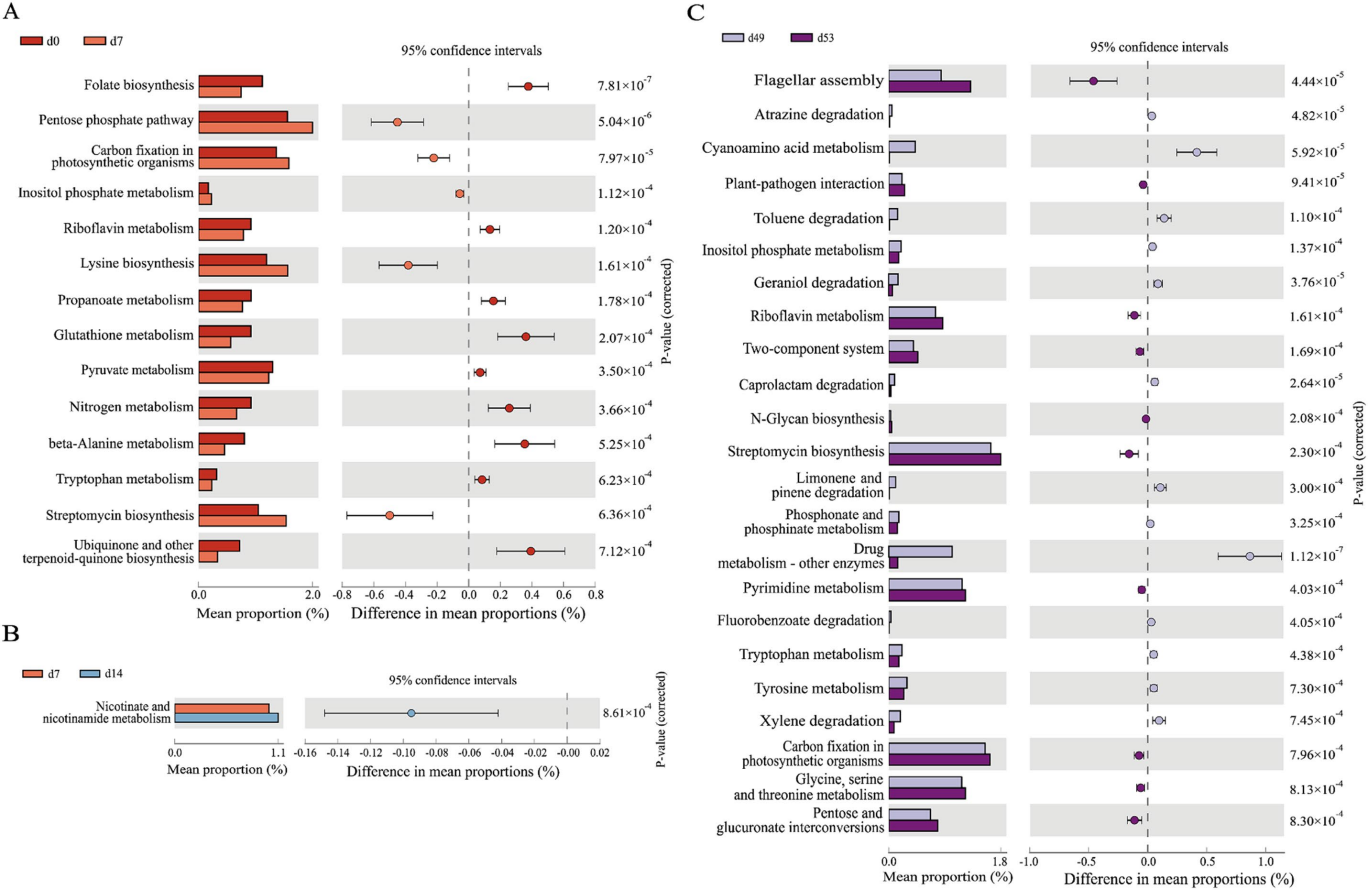
PICRUSt 2 functional prediction analysis. Prediction of the differential function in goat kids of fecal microbiota **(A)** between d0 and d7, **(B)** between d7 and d14, **(C)** between d49 and d53. The leftmost endpoint of each circle in the figure represents the 95% confidence interval lower limit of the mean difference, and the rightmost endpoint of the circle represents the 95% confidence interval upper limit of the mean difference. The group represented by the circle color is a group with a high mean. At the far right of the displayed results is the significance test *p*-value for pathway (*n* = 20, male kid = 10, female kid = 10).

The use of PICRUSt to forecast the functional attributes of 16 s rRNA sequencing data leveraging the Kyoto Encyclopedia of Genes and Genomes (KEGG) database has yielded results. Figure 5 encapsulated the three groups of KEGG pathways that we were more concerned about, which have extremely significant differences (*p* < 0.01) in abundance at various time points. Notably, the pathways exhibiting substantial divergence (*p* < 0.01) between the two compared groups on d0 and d7 were predominantly associated with substance synthesis and metabolic processes. This includede 14 pathways, such as those involved in folate biosynthesis, the pentose phosphate pathway, inositol metabolism, riboflavin metabolism, and lysine biosynthesis, as depicted in Figure 5A. The pathway with significant differences (*p* < 0.01) between the two groups on d7 and d14 was nicotinate and nicotinamide metabolism (Figure 5B). Among the KEGG pathways predicted by goat kids before and after weaning (d49 and d53), there were 23 pathways with significant differences (*p* < 0.01). As represented in Figure 5C, including drug metabolism - other enzymes, caprolactam degradation, cyanoamino acid metabolism, atrazine degradation, geraniol degradation, among others.

## 4 Discussion

In recent years, a wealth of microbiome research has shed light on the pivotal role that the gut microbiota plays in the health of its host. It has become increasingly clear that alterations to the gut microbiota can have a direct impact on the host’s overall health and their susceptibility to various diseases (Sonnenburg et al., 2016). Currently, a substantial body of research examined the fluctuations in gut microbiota in animals during the periods preceding and following illness. However, there was a notable scarcity of studies that concentrate on the alterations and the process of colonization of gut microbiota specifically in goat kids, from the time of their birth through the weaning phase. In the present investigation, we conducted a systematic analysis of the fecal microbiota composition and its variations in goat kids from birth to the weaning stage. Our findings revealed that the microbial community in Tianfu goat kids undergoes distinct and sequential phases of development throughout this period.

A rich and highly diverse gut microbiota is widely recognized as beneficial to the host’s health and serves as one of an indicator of a mature gut microbiota, thereby suggesting that the hosts were in optimal health (Li et al., 2012; Wang et al., 2018). In this study, microbial diversity significantly increased from d21 onwards. After weaning (d53), diversity decreased and tended to stabilize. These could be attributed to the combined effects of age and dietary changes. The fecal microbiota of sheep and goats are predominantly composed of the phyla *Firmicutes, Proteobacteria* and *Bacteroidetes* (Belanche et al., 2019; Wang et al., 2018), a pattern that is similarly observed in piglets, cattle and monkeys (Alipour et al., 2018; Looft et al., 2014; Yasuda et al., 2015). Our research has identified a phased fluctuation in the relative abundance of the *Firmicutes* phylum. Initially, it was at its lowest on d0 (21.66%). Following this, we observed a continuous alteration from d7 to d35 (46.42–74.36%). From d42 (60.20%) to before weaning (d49, 61.09%), the relative abundance gradually stabilizes and increases after weaning (d53, 78.15%) before stabilizing. Meanwhile, on the day of goat kids birth (d0), the abundance of *Proteobacteria* is highest (72.34%). Afterwards, it was quickly replaced by *Firmicutes* and *Bacteroidetes*, especially after weaning. The changing trend of *Proteobacteria* has also appeared in the intestines of the Shaanbei White Cashmere Goat and human infants (Fallani et al., 2011; Koenig et al., 2011; Li et al., 2019). This series of microbial changes is highly likely to indicate the dynamic properties of the fecal microbiome of juvenile ruminants and its relationship with the developmental stage of the host.

Significant shifts in the relative abundance of fecal microbiota were observed in goat kids within 14 days after birth, likely attributable to the underdeveloped structure or incomplete physiological function of the gastrointestinal tract during this early stage. These fluctuations tended to stabilized by the 21st day after birth. Before weaning the goat kids, the relative abundance of the main microbial community at the genus level tended to stabilize from d21 to d49. Until the goat kids were weaned and no longer consume breast milk and milk substitutes, the composition of the gut microbiota changed again after consuming solid feed and plant fiber. The changes in fecal microbiota are not only related to diet, but also closely related to the development of the rumen. The rumen function gradually improves, the intake of solid feed increases, and the types and quantities of microorganisms involved in cellulose degradation and digestion continue to increase (Ferretti et al., 2018; Rey et al., 2014). For ruminants, the colonization of beneficial bacterial strains plays a crucial role in aiding their adaptation to environmental and dietary changes. The colonization of beneficial bacteria in the gastrointestinal tract occurs in the early stages of life, which is crucial for their potential function in adulthood.

We used LEfSe to reveal the differential microbiota at three important time points (d0 vs. d7, d7 vs. d14, d49 vs. d53) in this experiment, which helps to better understand the colonization of fecal microbiota in early life stages of ruminants. The feces of newborn goat kids found to contain *Shigella* from the *Enterobacteriaceae* family, which is present in the feces of human infants as well (Bäckhed et al., 2015). Related studies have found that *Shigella* can inhibit or affect various signaling pathways of host cells by secreting effector proteins, in order to evade the host’s immune defense (Li et al., 2021; Zychlinsky et al., 1992). Zhu et al. detected *Shigella* from yak diarrhea feces and found that the isolated *Shigella* carried multiple virulence genes, posing a potential threat to the host and in severe cases, could lead to host death (Zhu et al., 2018). On the d7, the bacterial biomarkers in goat kids were *Pulicaecorum* and *Fragilis*, which may be related to breastfeeding rich in protein, fat, and lactose. As a strict anaerobic bacterium, *Pullicaecorum* is capable of producing butyric acid, which strengthens the integrity of the intestinal epithelial barrier and diminishes intestinal permeability (Geirnaert et al., 2017). This action is crucial for preventing the invasion of pathogens and harmful substances into the body. Additionally, *Pullicaecorum* appears to play a role in modulating the composition of the gut microbiota, thereby contributing to a balanced and healthy intestinal environment (Chang et al., 2020). Meanwhile, *Fragilis* could prevent *Clostridium difficile* infection in mouse models by restoring the intestinal barrier and regulating the microbiome (Deng et al., 2019). The early colonization of beneficial bacterial strains in ruminants helps to better adapt to environmental changes and can also better resist the invasion of some pathogenic bacteria in the intestine.

After weaning, goat kids experience changed in both their diet and living conditions. They ceased to consume breast milk and begun to live independently from their mothers. The corresponding composition of fecal microbiota has also changed. From the research results, we found that the levels of *Ruminococcaceae* and *Bacteroidetes* in the intestines of weaned goat kids (d53) were significantly higher than those in sucking kids at 49 days old. *Ruminococcaceae* and *Bacteroidetes* were of great significance for gut barrier function and maintaining the balance of gut microbiota. *Ruminococcaceae* mainly existed in the biofilm of the colon mucosa in healthy individuals (De Weirdt and Van de Wiele, 2015). The decrease in its abundance was associated with many inflammatory bowel diseases and *Clostridium difficile* infections (Antharam et al., 2013; Joossens et al., 2011; Morgan et al., 2012). Meanwhile, research has found that the *Ruminococcaceae* family primarily maintains intestinal health by producing butyrate and short chain fatty acids (SCFAs). These SCFAs were essential carbon and energy sources for colon epithelial cells (Wong et al., 2006). When the colon lacks SCFAs, it may lead to colonic mucosal dysfunction, leading to host diarrhea (Young and Schmidt, 2004). Interestingly, some bacteria in the phylum *Bacteroidetes* also produced SCFAs that could affect the brain and intestines (Mirzaei et al., 2021). Related studies have found that changes in the host’s environmental factors (such as diet) to some extent determine the content of *Bacteroidetes* (Goodrich et al., 2016; Goodrich et al., 2014). When the relative content of multiple microbial communities is maintained at a certain proportion, it is more conducive to maintaining the relative stability of the host’s intestine, which can to some extent reduce the probability of the host’s illness.

In summary, we found that the types of fecal microbiota in goat kids gradually increase after birth and tend to stabilize after weaning. This change is mainly related to the age and dietary changes of goat kids, and helps them enhance their material metabolism ability. Although further exploration and validation of the relevant mechanisms are needed through more omics sequencing, our results provide data support for a better understanding of the role of microbiota in young ruminants.

## 5 Conclusion

In this study, we profiled the changes in fecal microbiota of healthy Tianfu goat kids from birth to weaning. According to the trend of fecal microbiota changed, it was divided into three stages. The trend of fecal microbiota changes was closely related to the development of the GIT and the composition of diet. Our findings provide new insights to the understanding the colonization of fecal microbiome in Tianfu goats. Further study is need to assess the benefit microbiota and utilize in improving the health and performance of young ruminants.

## Data Availability

The raw data has been uploaded to China National Center for Bioinformation (CRA017094).
