# Supplementary material for: Colonization profiles of gut microbiota in goat kids from neonatal to weaning period
Source: Front Microbiol. 2024 Oct 1;15:1467205. doi: 10.3389/fmicb.2024.1467205 (PMC11473314; doi:10.3389/fmicb.2024.1467205)
Supplement: Supplementary file 1 [file Data_Sheet_1.PDF]

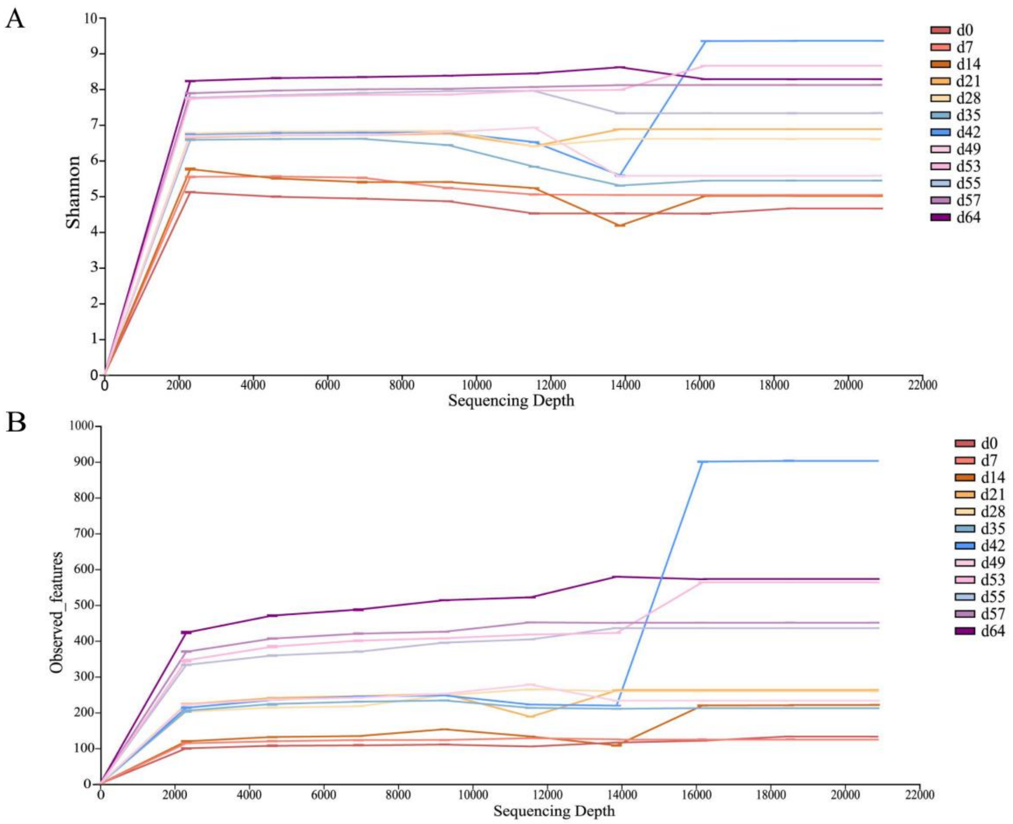

Supplementary Figure 1 Rarefaction analyses of all sampling groups.

(A) Rarefaction curves of all sampling groups based on the Shannon index. **(B)** Rarefaction curves of all sampling groups based on the observed.

Supplementary Table 1.

Quality and diversity index of microbial sequencing in goat kids feces

Sample	Raw_Tags	Clean_Tags	Effective (%)	Q20%	Q30%	GC%	ASV	Shannon	Chao1
TF2015-0	62023	58269	93.95	97.73	93.56	53.64	9757	2.9007	40
TF2046-0	87920	83063	94.48	97.81	93.3	54.54	2716	3.6729	51
TF2009-0	82234	75635	91.98	97.96	94.03	54.82	17595	2.7216	34
TF2028-0	85452	79085	92.55	97.29	92.21	53.27	5237	3.1162	43
TF2042-0	83517	73659	88.2	98.21	94.66	53.74	9425	3.4299	64
TF2044-0	84499	75650	89.53	98.28	94.83	53.63	11022	3.4111	51
TF1993-0	80030	75104	93.84	97.92	93.76	54.08	12598	2.8510	32
TF2011-0	80925	75175	92.89	98.08	94.35	53.91	15091	3.1311	37
TF2048-0	84869	79633	93.83	98.09	94.28	53.8	7767	3.4147	37
TF2038-0	87646	80501	91.85	97.63	93.35	52.94	5553	3.9150	70
TF2036-0	81847	64928	79.33	98.08	94.31	54.01	5792	3.4640	60
TF1987-0	85086	72836	85.6	98.12	94.41	53.18	8280	3.1451	37
TF2040-0	86532	67359	77.84	98.05	94.3	53.29	9610	3.2532	45
TF2007-0	83560	78463	93.9	98.12	94.48	54.37	19241	2.7778	50
TF2066-0	80667	75334	93.39	97.99	94.02	53.85	15148	2.9143	55
TF1997-0	85717	80935	94.42	98.18	94.68	54.62	15714	2.4337	27
TF1999-0	84466	78632	93.09	95.15	86.98	54.86	13599	2.0657	18
TF2001-0	84611	79636	94.12	98.07	94.37	54.04	19247	2.7259	41
TF2060-0	87521	81254	92.84	97.99	93.83	53.9	10133	2.7345	25
TF1995-0	83025	78559	94.62	98.15	94.59	54.54	14900	2.4808	25
TF2015-7	51366	47705	92.87	97.23	92.42	53.3	4248	3.8714	65
TF2046-7	85600	76018	88.81	98.04	94.23	54.11	15359	3.0478	61.5
TF2009-7	87966	68278	77.62	97.57	93.05	52.13	13345	3.3644	57
TF2028-7	85696	69081	80.61	97.57	92.79	54.21	6132	3.2208	43
TF2042-7	86822	77959	89.79	97.88	93.83	53.18	10589	3.2894	55
TF2044-7	80288	70958	88.38	97.91	93.93	53.99	15948	3.0473	43
TF1993-7	80999	75164	92.8	98.08	93.99	54.99	15772	2.3753	42
TF2011-7	81380	74781	91.89	97.8	93.52	54.02	6167	3.3229	61
TF2048-7	82698	71478	86.43	98.01	94.1	53.58	8027	3.2799	58
TF2038-7	87998	75576	85.88	97.96	93.92	53.16	7221	3.4463	61
TF2036-7	85383	67993	79.63	97.79	93.57	53.49	8883	3.5236	62
TF1987-7	86471	71536	82.73	97.88	93.87	53.91	13847	3.4676	66
TF2040-7	81160	66705	82.19	97.89	93.76	52.9	7509	3.6959	59
TF2007-7	81694	73597	90.09	97.26	91.81	52.34	6826	3.3690	55
TF2066-7	82946	62993	75.94	97.95	93.94	53.67	5010	3.6966	66
TF1997-7	85948	62958	73.25	97.67	93.35	51.8	9269	3.7306	53
TF1999-7	81302	62451	76.81	97.99	94.07	51.48	8663	3.7488	74

TF2001-7	82489	68488	83.03	98.02	94.09	52.76	7570	4.0485	80
TF2060-7	84703	75911	89.62	97.46	92.55	53.3	8721	2.9958	46
TF1995-7	81043	74325	91.71	97.91	93.83	52.56	11360	3.1226	48
TF1987-14	61691	57951	93.94	97.4	92.59	53.77	6090	3.2713	51
TF1993-14	85607	76098	88.89	97.24	92.2	51.75	11340	3.1998	50
TF1995-14	87588	79047	90.25	97.86	93.68	54.64	3201	3.6125	46
TF1997-14	83475	72538	86.9	94.96	86.61	54.26	3535	3.8704	57
TF1999-14	82754	68717	83.04	98.06	94.39	54.63	12222	3.4675	55
TF2001-14	86796	77661	89.48	97.93	93.89	52.41	10134	2.8545	51
TF2007-14	81789	64968	79.43	98.08	94.31	54.25	12629	2.7884	39
TF2009-14	84485	72418	85.72	97.93	93.95	53.36	7557	2.9165	43
TF2011-14	86601	80410	92.85	98.04	94.12	54.39	20850	2.2390	14
TF2015-14	87765	67179	76.54	98	94.21	53.81	9595	3.1063	43
TF2028-14	85483	68575	80.22	97.29	92.27	52.35	10502	3.3558	64
TF2036-14	87160	68706	78.83	97.47	92.74	52.51	9242	3.1844	40
TF2038-14	87577	77409	88.39	98.04	94.13	54.93	18880	2.4174	34
TF2040-14	55832	52610	94.23	97.25	92.29	53.37	6620	3.6869	53
TF2042-14	50078	46518	92.89	97.74	93.71	52.1	4577	2.9327	26
TF2044-14	58065	54709	94.22	97.54	92.72	53.72	6273	2.8397	31
TF2046-14	83710	77843	92.99	98.03	94.25	54.53	9347	3.1696	43
TF2048-14	87711	78407	89.39	97.35	92.5	54.48	15466	2.4387	29
TF2060-14	86182	72046	83.6	97.67	93.26	54.27	4130	3.7107	54
TF2066-14	82159	77643	94.5	98.03	94.19	54.02	12006	2.7419	32
TF1987-21	85448	72655	85.03	98.07	94.18	54.21	11762	3.7153	83
TF1993-21	85967	71538	83.22	98.24	94.82	52.05	10981	4.0073	94
TF1995-21	81253	61582	75.79	98.13	94.69	52.54	11117	4.3506	116
TF1997-21	86394	64711	74.9	94.52	85.45	52.97	7261	2.2718	10
TF1999-21	87155	80353	92.2	98.23	94.67	53.07	12711	3.1473	47
TF2001-21	83126	74003	89.03	97.26	91.93	52.05	12804	3.5465	66
TF2007-21	52969	49959	94.32	98.05	94.26	53.14	7328	3.9473	67
TF2009-21	58742	54905	93.47	97.91	94.1	55.26	9314	3.7006	74
TF2011-21	87598	65822	75.14	98.02	94.33	53.23	10703	4.2902	122
TF2015-21	87729	71767	81.81	98.15	94.56	54.01	14192	4.1993	125
TF2028-21	80143	71170	88.8	98.02	94.02	54.08	10659	2.7353	61
TF2036-21	87225	69546	79.73	98.27	94.9	52.53	9597	3.7630	79
TF2038-21	86876	69131	79.57	98.28	94.97	53.04	10795	3.8134	111
TF2040-21	86340	78393	90.8	98.2	94.65	51.79	11251	2.9681	47
TF2042-21	82547	70228	85.08	98.33	95.06	52.86	10176	4.3204	108
TF2044-21	81555	63857	78.3	97.48	92.68	53.16	9769	4.6743	139
TF2046-21	82223	63134	76.78	98.04	94.26	54.86	10722	3.6831	77
TF2048-21	82293	58278	70.82	97.45	92.63	53.45	12293	3.8328	80
TF2060-21	85796	75596	88.11	98.29	94.99	52.98	10947	3.9767	87
TF2066-21	83323	74873	89.86	98.35	95.14	53.18	10875	3.3052	70
TF1987-28	85494	68250	79.83	94.84	86.38	53.39	9071	3.7683	49

TF1993-28	82121	69055	84.09	97.99	94.13	53.25	14234	4.0562	105
TF1995-28	86058	71234	82.77	97.92	93.97	53.41	15085	4.4075	155
TF1997-28	87478	66200	75.68	97.81	93.71	52.39	11118	4.2837	123
TF1999-28	83206	64840	77.93	95.94	88.9	53.34	10358	4.2994	109
TF2001-28	81168	65611	80.83	97.92	93.95	53.45	15634	3.7794	123
TF2007-28	59688	55920	93.69	97.32	92.47	53.57	9239	4.2523	114
TF2009-28	82580	59129	71.6	98.06	94.4	56.02	13593	3.5413	96
TF2011-28	53740	48119	89.54	94.2	85.17	53.31	7141	3.9301	65
TF2015-28	87485	66484	75.99	97.86	93.86	53.37	9113	3.1424	41
TF2028-28	57230	53550	93.57	98.02	94.44	53.9	7845	3.8162	85
TF2036-28	80390	67307	83.73	97.92	93.96	54.51	12882	3.6623	108
TF2038-28	82820	69964	84.48	97.83	93.78	52.76	11340	4.1642	118
TF2040-28	80947	70381	86.95	96.79	90.78	52.33	9998	3.4490	48
TF2042-28	80662	69717	86.43	97.49	92.75	53.62	7642	3.2857	36
TF2044-28	59172	55453	93.71	97.67	93.44	52.58	7671	4.3346	108
TF2046-28	82924	68184	82.22	97.52	92.68	55.5	14569	3.5962	78
TF2048-28	86266	71588	82.99	97.31	92.34	52.87	10027	3.7829	59
TF2060-28	86196	78243	90.77	97.98	94.11	52.8	12158	4.4170	122
TF2066-28	84920	75599	89.02	96.9	91.11	52.12	7151	3.3736	48
TF1987-35	87365	76325	87.36	97.46	92.81	52.41	9849	3.8931	66
TF1993-35	80655	69987	86.77	95.86	88.62	54.49	12547	3.3870	72
TF1995-35	83758	69419	82.88	98.08	94.34	54.15	10138	3.1389	39
TF1997-35	80383	65070	80.95	96.46	90.25	53.41	10865	4.3172	102
TF1999-35	84097	62474	74.29	97.52	92.79	54.21	8855	4.3888	107
TF2001-35	87835	62743	71.43	97.56	93.07	53.84	9855	4.3364	120
TF2007-35	60417	56798	94.01	97.44	92.76	53.41	10896	3.6450	89
TF2009-35	85799	65920	76.83	98.11	94.58	55.55	13678	3.3494	91
TF2011-35	87939	69412	78.93	98.05	94.32	53.8	9634	3.9408	80
TF2015-35	80320	65890	82.03	98.28	95	57.05	14926	2.9189	66
TF2028-35	52085	48387	92.9	97.73	93.66	53.33	7463	3.6152	61
TF2036-35	81593	67232	82.4	96.81	90.84	54.61	12995	3.5164	86
TF2038-35	82345	67353	81.79	97.76	93.57	54.49	12465	4.1112	101
TF2040-35	81976	68797	83.92	98.06	94.26	52.57	10740	4.3062	102
TF2042-35	84933	62474	73.56	97.63	93.15	52.65	8823	3.4365	43
TF2044-35	84868	74021	87.22	97.54	92.88	52.5	11113	4.1223	97
TF2046-35	81361	69486	85.4	98.21	94.78	56.79	16221	3.1185	81
TF2048-35	84263	66912	79.41	97.31	92.38	52.72	7717	3.7639	57
TF2060-35	85550	71301	83.34	98.11	94.4	53.42	10065	4.3649	109
TF2066-35	82612	73796	89.33	96.37	89.66	54.05	11451	3.0782	48
TF1987-42	82810	73144	88.33	95.61	88.07	53.37	9884	3.1588	48
TF1993-42	87798	67728	77.14	98.03	94.28	53.37	10561	3.6527	85
TF1995-42	81454	69304	85.08	97.03	91.31	53.77	9467	3.4424	44
TF1997-42	85091	67707	79.57	98.07	94.37	53.38	10478	4.4565	107
TF1999-42	80832	70488	87.2	97.51	92.85	53.67	10457	3.8719	71

TF2001-42	82740	71147	85.99	97.56	92.96	53.11	9939	4.0360	67
TF2007-42	82673	66693	80.67	97.34	92.23	53.63	10782	3.5338	72
TF2009-42	85621	72618	84.81	97.86	93.72	56.18	14095	2.6883	43
TF2011-42	84486	69418	82.17	97.61	93.19	53.2	12484	3.8694	66
TF2015-42	87670	76934	87.75	98.19	94.57	54.66	10236	3.5704	66
TF2028-42	84705	69576	82.14	97.64	93.34	56.7	10280	2.6224	51
TF2036-42	87668	71791	81.89	98.24	94.81	53.76	13476	3.8791	85
TF2038-42	83918	69255	82.53	98.22	94.81	53.54	10882	3.9977	71
TF2040-42	86046	76598	89.02	98.04	94.16	53.35	13226	3.2118	35
TF2042-42	83917	73162	87.18	97.74	93.46	52.79	20668	2.4284	13
TF2044-42	80199	71943	89.71	97.17	91.55	53.76	15113	2.7017	39
TF2046-42	80182	62456	77.89	97.82	93.44	57.25	13409	2.8440	56
TF2048-42	87808	71065	80.93	98.23	94.72	51.27	9848	2.9513	50
TF2060-42	85337	75216	88.14	96.76	90.62	53.51	10058	3.6272	49
TF2066-42	87822	73159	83.3	97.96	93.97	52.98	10145	3.4858	50
TF1987-49	83835	72424	86.39	98.28	94.94	52.86	9543	3.4742	58
TF1993-49	80438	68562	85.24	97.55	92.85	53.34	8876	3.2560	46
TF1995-49	80938	72683	89.8	98.23	94.59	53.7	13723	2.4870	38
TF1997-49	83475	68549	82.12	97.8	93.58	53.32	11500	4.0072	88
TF1999-49	84773	68095	80.33	98.17	94.68	52.74	11916	4.4697	111
TF2001-49	81927	63814	77.89	98.14	94.66	53.31	12872	3.8637	105
TF2007-49	87232	74413	85.3	96.87	91.38	52.29	8127	3.3912	53
TF2009-49	86479	73808	85.35	98.33	95.08	57.06	14012	2.8136	59
TF2011-49	85852	73106	85.15	96.97	91.69	53.64	12613	3.2055	48
TF2015-49	81600	63077	77.3	98.26	94.88	53.05	11550	3.0331	54
TF2028-49	86552	72382	83.63	96.96	91.09	57.25	8610	3.1802	41
TF2036-49	87930	75145	85.46	97.81	93.51	52.8	11669	4.2359	98
TF2038-49	86096	72911	84.69	98.27	94.89	52.89	8413	3.3572	74
TF2040-49	81487	71419	87.64	97.4	92.37	51.99	11321	3.6772	51
TF2042-49	87664	75560	86.19	95.05	86.65	52.68	11405	3.6202	52
TF2044-49	83759	73707	88	97.33	92.45	52.53	10704	3.5133	58
TF2046-49	87562	69408	79.27	98.31	95.01	56.32	13925	3.1571	72
TF2048-49	85718	72095	84.11	97.69	93.08	53.2	13599	3.0957	31
TF2060-49	82974	75226	90.66	97.27	92.33	51.51	10184	3.5364	55
TF2066-49	86636	76174	87.92	98.27	94.93	53.02	10781	3.5042	72
TF1987-53	86119	72789	84.52	98.21	94.75	53.01	10821	3.7890	82
TF1993-53	82103	69041	84.09	98.26	94.73	51.77	6461	2.5020	33
TF1995-53	85467	74330	86.97	97.79	93.47	53.45	15345	2.7232	39
TF1997-53	83876	69919	83.36	98.12	94.5	52.56	12470	3.9467	105
TF1999-53	83770	71581	85.45	98.1	94.45	52.62	12466	4.2980	97
TF2001-53	81993	67525	82.35	93.02	82.38	52.93	12389	4.0467	64
TF2007-53	87393	72707	83.2	98.12	94.47	52.19	11545	3.5493	84
TF2009-53	84228	69770	82.83	95.12	86.86	53.7	12961	3.9912	68
TF2011-53	82375	68580	83.25	98.08	94.37	52.85	9516	4.3104	91

TF2015-53	86461	70655	81.72	97.59	92.84	53.31	12842	3.7704	64
TF2028-53	87292	75308	86.27	97.19	91.95	52.89	15624	3.3989	45
TF2036-53	85004	71648	84.29	98.07	94.39	53.63	11182	4.2347	92
TF2038-53	86102	73227	85.05	97.93	93.81	52.34	14369	3.8166	77
TF2040-53	83821	70394	83.98	97.63	92.88	53.36	10304	3.8029	69
TF2042-53	84209	70927	84.23	95.94	88.52	52.43	11468	3.1070	31
TF2044-53	84742	73690	86.96	97.92	93.93	52.24	15219	3.5577	74
TF2046-53	81624	70148	85.94	97.94	94.03	53.56	16188	4.0593	75
TF2048-53	84581	70433	83.27	98.1	94.42	52.32	10557	3.7965	68
TF2060-53	85793	74092	86.36	97.47	92.81	51.98	10720	3.1941	38
TF2066-53	84089	69372	82.5	97.68	93.3	52.05	9602	3.1120	28
TF1987-55	80425	63277	78.68	93.58	83.55	52.78	8472	3.6842	46
TF1993-55	84074	73679	87.64	97.31	91.97	53.1	12225	3.8162	71
TF1995-55	84370	69978	82.94	97.1	92.08	54.45	8624	3.3443	47
TF1997-55	86904	73062	84.07	98.03	94.14	52.88	13643	3.5067	90
TF1999-55	83369	72306	86.73	97.96	93.78	53.02	11843	3.9257	82
TF2001-55	84908	70469	82.99	98.01	94.16	52.95	14456	3.6644	122
TF2007-55	85218	72382	84.94	95.3	87.22	53.54	9637	3.9423	65
TF2009-55	82870	73301	88.45	97.57	92.94	53.94	10651	3.7250	66
TF2011-55	86813	64063	73.79	90.03	77.26	53.11	6244	2.8208	20
TF2015-55	84635	66829	78.96	97.85	93.78	53.12	10694	3.6431	63
TF2028-55	83760	72948	87.09	97.21	91.89	52.5	15385	2.6703	19
TF2036-55	82244	73425	89.28	96.74	90.53	53.37	11659	3.9081	64
TF2038-55	87958	79315	90.17	97.99	93.96	53.84	11081	2.2961	35
TF2040-55	82408	66327	80.49	98.05	94.31	53.53	8549	3.7771	68
TF2042-55	82451	67275	81.59	97.9	93.9	52.84	8135	3.4088	48
TF2044-55	82675	70619	85.42	97.6	93.12	52.65	11363	3.7160	55
TF2046-55	85034	74477	87.58	97.3	92.07	52.67	13074	3.6856	57
TF2048-55	82757	69917	84.48	97.66	93.01	52.96	11380	4.0393	74
TF2060-55	81706	69803	85.43	98.2	94.66	52.91	10874	4.0320	75
TF2066-55	81900	69660	85.05	97.37	92.21	52.76	12051	3.1044	30
TF1987-57	86512	73567	85.04	98.09	94.32	52.9	11193	3.9900	90
TF1993-57	85599	78265	91.43	98	93.84	53.26	11610	3.2157	47
TF1995-57	86241	72029	83.52	97.87	94.02	55.6	10997	3.6418	64
TF1997-57	80030	67646	84.53	94.7	85.84	53.02	10241	3.6141	47
TF1999-57	82154	71198	86.66	97.34	92.09	53.59	11177	3.5801	59
TF2001-57	83635	71026	84.92	97.75	93.47	53.9	10880	3.5096	45
TF2007-57	87081	73628	84.55	97.94	93.93	52.53	12334	3.9770	82
TF2009-57	86587	68357	78.95	98.13	94.46	52.47	10184	3.5519	62
TF2011-57	82732	70772	85.54	97.76	93.39	52.74	11057	3.8850	67
TF2015-57	86518	69245	80.04	98.27	94.81	53.05	12252	3.1822	78
TF2028-57	86531	73219	84.62	97.92	93.78	53.12	14059	3.8021	57
TF2036-57	84022	77118	91.78	97.89	93.53	53.04	14676	3.5914	58
TF2038-57	83035	69414	83.6	98.05	94.26	52.94	13776	3.3425	41

TF2040-57	83088	72282	86.99	98.1	94.35	52.7	11553	4.0952	89
TF2042-57	80352	62094	77.28	98.03	94.28	52.72	11483	3.8595	66
TF2044-57	86291	75646	87.66	95.62	87.94	53.08	11395	3.3683	34
TF2046-57	85081	72313	84.99	97.73	93.31	53.2	14324	2.8477	28
TF2048-57	84261	71139	84.43	97.84	93.43	52.9	11428	4.0754	77
TF2060-57	86828	72300	83.27	97.32	92.13	52.64	11857	3.6123	48
TF2066-57	81844	73264	89.52	98.1	94.3	52.62	9680	3.5851	58
TF1987-64	83416	68739	82.41	95.49	87.74	53.08	9181	3.5694	48
TF1993-64	86114	74623	86.66	97.64	92.85	54.02	16506	3.1913	48
TF1995-64	83956	75149	89.51	97.65	93.32	53.17	15023	2.8026	21
TF1997-64	85245	71451	83.82	98.12	94.45	53.82	10360	4.2170	84
TF1999-64	85515	73483	85.93	97.81	93.33	53	11609	3.8074	79
TF2001-64	81067	65277	80.52	98.01	94.02	53.26	11494	3.8981	63
TF2007-64	81486	61819	75.86	94.61	85.76	53.3	9212	3.7483	48
TF2009-64	87611	75343	86	98.21	94.73	52.47	15117	3.9337	71
TF2011-64	85651	73589	85.92	93.62	83.59	53.98	12374	3.8405	53
TF2015-64	61933	58954	95.19	98.13	94.68	52.94	8578	3.5007	58
TF2028-64	83222	74138	89.08	98.19	94.72	53.68	15795	3.4394	37
TF2036-64	80509	71016	88.21	97.62	92.88	52.69	12428	3.8862	60
TF2038-64	83296	72798	87.4	98.16	94.52	53.3	10625	3.9430	73
TF2040-64	84082	72137	85.79	98.18	94.67	52.76	13988	3.5677	56
TF2042-64	83951	74302	88.51	98.14	94.51	53.16	12222	3.7513	64
TF2044-64	86027	75042	87.23	96.31	89.6	53.22	12077	2.8899	23
TF2046-64	85641	74774	87.31	97.95	93.77	53.12	13581	3.7990	59
TF2048-64	84070	74307	88.39	97.6	92.73	53.07	13551	3.4739	62
TF2060-64	86232	74253	86.11	98.2	94.61	53.16	12492	4.0347	81
TF2066-64	84155	71968	85.52	98.25	94.82	52.9	12116	3.6385	49

Supplementary Table 2.

The abundances of major bacterial genera in the rumen of goat kids from birth to d64.

Items	0d	7d	14d	21d	28d	35d	42d	49d	53d	55d	57d	64d
Shigella	70.87%	0.00%	0.00%	34.71%	2.72%	0.93%	0.20%	0.37%	1.80%	1.60%	1.27%	1.93%
	81.88%	8.55%	0.00%	26.56%	21.47%	58.66%	4.77%	1.13%	3.17%	1.42%	2.26%	20.44%
	79.39%	10.33%	0.00%	4.99%	14.58%	43.72%	1.82%	1.03%	1.01%	0.88%	1.25%	0.48%
	0.77%	0.00%	0.00%	1.85%	25.59%	6.14%	0.38%	0.25%	1.16%	1.48%	1.31%	1.82%
	56.86%	0.60%	15.27%	25.81%	17.17%	5.86%	0.00%	0.44%	0.91%	1.68%	1.36%	1.03%
	86.68%	0.65%	2.64%	22.72%	47.44%	7.18%	4.29%	0.13%	0.83%	1.37%	0.85%	1.06%
	75.89%	0.00%	70.94%	14.77%	25.88%	4.97%	0.23%	1.44%	1.31%	1.45%	1.86%	4.79%
	52.65%	0.00%	2.29%	3.86%	1.50%	0.82%	0.32%	0.63%	1.64%	1.92%	1.57%	0.79%
	0.00%	0.00%	62.66%	8.63%	27.64%	2.56%	0.00%	6.99%	1.52%	0.00%	1.53%	0.84%
	69.20%	0.18%	1.81%	20.86%	38.63%	0.55%	2.02%	0.50%	1.40%	1.74%	1.13%	1.05%
	94.35%	0.00%	3.32%	2.81%	4.35%	26.57%	0.74%	0.00%	0.51%	1.60%	1.17%	1.01%
	60.94%	2.81%	10.00%	22.71%	47.57%	51.01%	0.11%	0.22%	0.21%	1.76%	1.29%	1.24%
	57.58%	0.00%	0.81%	27.79%	1.58%	16.12%	0.25%	1.11%	0.98%	0.59%	2.58%	1.19%
	80.46%	0.00%	25.35%	58.87%	0.73%	2.14%	23.74%	0.00%	1.14%	2.13%	1.56%	0.71%
	68.33%	0.16%	7.54%	2.01%	0.88%	1.94%	0.00%	0.70%	0.99%	2.86%	1.19%	1.21%
	75.02%	33.97%	60.10%	5.06%	2.76%	0.58%	0.00%	1.09%	0.00%	1.91%	0.83%	0.88%
	79.74%	0.39%	11.46%	2.00%	8.17%	0.52%	0.20%	1.01%	0.89%	1.52%	0.00%	0.84%
	77.17%	0.00%	85.56%	6.32%	6.68%	0.79%	1.11%	1.01%	0.85%	1.67%	2.09%	1.85%
	75.32%	0.37%	0.00%	9.20%	5.19%	2.40%	1.09%	0.98%	1.02%	1.58%	0.74%	0.89%
	74.96%	5.77%	52.11%	3.51%	0.00%	0.95%	1.46%	0.22%	0.49%	2.17%	1.57%	0.78%

Items	0d	7d	14d	21d	28d	35d	42d	49d	53d	55d	57d	64d
	0.00%	1.83%	1.30%	6.74%	1.94%	12.62%	7.73%	28.02%	0.55%	0.00%	0.59%	0.61%
	0.35%	10.67%	2.35%	1.62%	0.76%	3.76%	37.81%	30.43%	32.63%	1.33%	31.06%	0.41%
	0.02%	0.00%	3.16%	0.98%	0.94%	6.58%	16.33%	66.28%	46.33%	2.12%	0.27%	0.15%
	0.44%	0.67%	0.34%	0.56%	0.99%	5.88%	6.51%	6.07%	0.30%	0.44%	0.75%	0.87%
	1.49%	0.74%	1.41%	0.00%	0.83%	1.04%	8.60%	4.20%	0.90%	0.63%	1.31%	1.08%
	0.00%	0.87%	0.06%	0.60%	0.61%	0.57%	4.62%	2.09%	1.77%	0.80%	2.55%	0.03%
	0.91%	0.00%	0.16%	1.20%	3.10%	1.04%	1.02%	1.58%	3.01%	0.95%	0.39%	0.98%
	0.00%	0.80%	0.00%	1.30%	0.43%	0.68%	0.58%	0.86%	0.64%	0.77%	1.14%	1.24%
	0.00%	0.56%	1.77%	0.71%	1.81%	4.47%	0.82%	32.82%	0.47%	0.00%	0.80%	0.00%
	0.21%	0.38%	0.00%	0.62%	0.36%	0.71%	1.22%	1.00%	0.31%	0.00%	0.71%	1.79%
Akkermansia	0.43%	0.22%	0.43%	1.17%	1.04%	0.90%	1.22%	3.84%	0.79%	0.40%	0.14%	0.10%
	0.00%	0.00%	0.00%	1.29%	4.35%	5.58%	25.65%	16.22%	4.10%	1.00%	8.80%	0.30%
	1.12%	0.76%	1.53%	0.42%	2.32%	11.54%	11.16%	1.41%	0.73%	2.21%	1.20%	0.83%
	0.20%	41.94%	0.00%	0.98%	14.77%	4.97%	32.48%	1.43%	0.15%	1.47%	0.45%	0.49%
	0.55%	1.18%	1.16%	0.79%	11.76%	5.88%	3.26%	22.66%	1.02%	40.99%	1.80%	0.58%
	0.00%	0.48%	0.16%	1.38%	1.08%	9.56%	62.18%	26.46%	1.79%	1.15%	2.57%	2.75%
	0.16%	0.83%	0.00%	0.97%	0.59%	0.84%	0.54%	3.30%	0.38%	0.99%	20.53%	1.07%
	0.85%	1.03%	0.25%	1.08%	18.27%	11.08%	1.29%	0.66%	0.82%	1.36%	3.52%	0.35%
	0.84%	1.34%	9.66%	17.35%	5.83%	6.23%	18.87%	13.70%	1.06%	1.52%	0.90%	1.71%
	0.17%	0.00%	0.03%	0.43%	4.20%	55.10%	2.02%	39.62%	0.95%	6.25%	12.98%	1.15%

Items	0d	7d	14d	21d	28d	35d	42d	49d	53d	55d	57d	64d
Lactobacillus	2.86%	2.66%	2.61%	2.91%	4.14%	2.97%	3.91%	4.43%	1.31%	3.15%	1.69%	2.61%
	7.53%	9.99%	1.44%	2.11%	2.39%	2.00%	3.72%	1.30%	2.43%	2.25%	1.09%	1.91%
	1.77%	64.21%	5.03%	5.38%	3.28%	2.59%	2.12%	1.57%	0.34%	1.96%	1.82%	1.10%
	2.98%	2.84%	2.57%	5.08%	4.11%	5.13%	8.32%	2.57%	1.74%	1.53%	3.22%	2.17%
	6.18%	2.34%	4.83%	7.44%	4.80%	7.22%	25.89%	4.22%	1.55%	1.78%	1.37%	1.47%
	2.47%	0.73%	62.95%	43.91%	2.99%	6.29%	5.48%	2.77%	0.75%	1.89%	0.34%	1.80%
	2.14%	0.73%	1.49%	3.30%	3.52%	2.86%	2.58%	1.53%	2.21%	2.40%	1.54%	3.47%
	2.71%	2.29%	58.38%	3.53%	1.65%	2.36%	0.68%	1.05%	1.84%	2.57%	2.37%	1.38%
	3.01%	1.20%	0.41%	3.52%	3.06%	4.67%	1.42%	1.82%	1.49%	0.87%	2.21%	0.83%
	13.96%	1.34%	2.53%	2.67%	3.90%	1.54%	2.78%	1.68%	1.37%	1.25%	1.68%	1.55%
	0.43%	1.60%	63.42%	2.96%	5.19%	3.72%	2.66%	3.04%	0.65%	2.50%	0.24%	2.14%
	15.85%	3.65%	47.99%	2.77%	4.00%	3.33%	7.95%	3.22%	0.52%	1.92%	1.16%	0.41%
	5.81%	1.27%	0.78%	3.09%	5.39%	4.00%	2.52%	2.49%	1.73%	0.69%	1.46%	0.68%
	1.71%	1.40%	5.76%	1.82%	2.50%	3.82%	1.46%	2.64%	1.33%	1.92%	1.11%	1.65%
	3.94%	2.22%	5.11%	7.32%	2.58%	2.91%	0.56%	1.09%	2.08%	2.09%	1.92%	0.64%
	0.96%	23.54%	1.80%	5.32%	5.09%	4.57%	0.21%	1.61%	0.39%	1.78%	1.77%	0.67%
	1.34%	1.16%	24.90%	2.99%	3.84%	2.35%	2.92%	1.70%	1.46%	3.88%	1.40%	0.62%
	1.50%	1.58%	1.24%	3.81%	4.48%	3.84%	4.09%	1.92%	1.45%	2.77%	1.21%	0.58%
	1.26%	1.53%	3.51%	3.26%	4.26%	4.28%	2.78%	4.93%	1.84%	1.66%	1.03%	1.63%
	0.97%	66.94%	0.25%	2.03%	5.03%	1.50%	2.63%	1.56%	0.80%	1.28%	1.12%	1.66%

Items	0d	7d	14d	21d	28d	35d	42d	49d	53d	55d	57d	64d
	0.81%	2.80%	1.28%	1.45%	1.32%	1.08%	0.62%	1.28%	0.26%	0.89%	0.00%	0.00%
	1.08%	7.49%	0.91%	1.27%	0.33%	0.59%	1.13%	0.22%	0.99%	0.46%	0.92%	0.92%
	0.74%	0.71%	3.25%	0.62%	0.90%	0.69%	0.68%	1.05%	0.35%	0.35%	1.38%	0.52%
	2.84%	1.89%	0.00%	1.94%	0.32%	0.58%	0.79%	1.62%	0.31%	0.48%	1.34%	1.38%
	2.09%	2.23%	1.27%	1.37%	0.50%	1.76%	0.98%	1.62%	0.64%	0.58%	0.36%	0.92%
	1.21%	1.54%	1.52%	0.82%	0.61%	1.26%	0.92%	1.24%	0.27%	0.24%	0.46%	1.33%
	1.16%	0.87%	0.89%	0.76%	1.38%	0.58%	1.03%	1.14%	0.40%	1.08%	0.71%	1.51%
	0.64%	2.19%	1.34%	45.18%	57.55%	54.85%	68.94%	73.22%	0.00%	0.17%	0.30%	0.49%
	0.69%	0.69%	0.45%	1.26%	1.21%	1.26%	0.99%	0.50%	0.85%	1.88%	0.77%	0.63%
Bifidobacteriu	1.92%	1.16%	1.00%	22.27%	1.56%	71.08%	0.96%	1.24%	0.31%	0.30%	0.35%	2.05%
	0.90%	1.97%	1.02%	1.32%	1.62%	2.13%	53.87%	22.77%	0.53%	0.58%	0.92%	0.00%
	0.12%	5.44%	0.77%	1.59%	1.20%	0.00%	1.08%	2.09%	0.00%	0.65%	0.34%	1.25%
	1.40%	1.21%	1.15%	1.21%	1.37%	0.84%	1.20%	2.74%	0.46%	0.42%	0.41%	1.40%
	0.71%	1.53%	1.47%	1.13%	0.00%	1.64%	0.74%	0.57%	0.09%	0.16%	0.95%	0.46%
	0.93%	2.14%	3.26%	0.33%	0.00%	0.45%	0.17%	0.49%	0.65%	0.33%	0.20%	1.31%
	0.66%	0.66%	1.88%	0.57%	2.80%	1.03%	0.00%	0.27%	0.57%	1.54%	0.00%	0.39%
	0.96%	1.72%	3.36%	30.70%	51.73%	65.74%	66.28%	55.71%	0.06%	0.31%	0.74%	1.01%
	0.76%	1.31%	0.81%	1.94%	0.85%	1.11%	2.18%	1.29%	0.33%	0.34%	0.48%	1.01%
	0.62%	1.53%	2.62%	1.03%	0.97%	1.37%	0.00%	0.26%	0.12%	0.00%	0.49%	0.33%
	0.46%	1.41%	0.78%	1.10%	0.74%	0.61%	0.68%	1.65%	0.00%	0.71%	0.51%	0.89%

Items	0d	7d	14d	21d	28d	35d	42d	49d	53d	55d	57d	64d
	0.67%	1.76%	0.92%	1.11%	4.88%	2.41%	0.50%	1.93%	4.93%	13.00%	6.91%	8.32%
	0.81%	4.84%	3.43%	0.78%	1.52%	0.98%	1.87%	2.92%	1.42%	7.75%	2.31%	5.32%
	0.03%	0.72%	3.50%	2.43%	2.54%	1.35%	3.61%	1.57%	1.55%	9.41%	7.86%	3.86%
	1.64%	0.84%	3.88%	2.29%	2.02%	1.09%	2.44%	2.73%	3.95%	5.57%	5.48%	4.12%
	5.50%	0.92%	3.40%	0.68%	2.50%	1.04%	2.69%	5.64%	8.29%	4.12%	13.20%	1.90%
	1.19%	0.75%	1.87%	0.33%	1.53%	2.57%	4.86%	3.71%	1.83%	4.96%	3.74%	2.84%
	1.06%	0.77%	0.58%	0.78%	3.09%	0.68%	2.55%	2.66%	4.68%	1.76%	16.05%	3.57%
	0.00%	2.73%	1.75%	0.48%	1.27%	0.93%	1.74%	0.65%	4.18%	5.28%	23.73%	5.32%
	1.14%	2.28%	1.30%	1.40%	0.77%	2.34%	8.54%	2.51%	4.99%	2.78%	5.62%	10.22%
Ruminococcus	1.38%	1.67%	0.45%	1.16%	4.57%	0.48%	4.38%	43.72%	4.69%	61.07%	39.44%	13.77%
	0.00%	0.92%	2.31%	1.48%	0.65%	1.59%	2.82%	11.55%	2.50%	17.29%	4.23%	5.99%
	1.68%	4.07%	1.67%	5.62%	3.89%	2.76%	6.61%	1.72%	1.10%	3.61%	3.20%	5.98%
	1.03%	3.62%	2.27%	1.33%	4.58%	0.60%	6.50%	2.69%	9.57%	4.40%	2.68%	5.52%
	0.52%	1.56%	0.00%	0.58%	3.68%	5.91%	1.82%	1.93%	2.63%	2.85%	4.90%	4.95%
	1.00%	3.27%	2.29%	1.37%	5.08%	3.71%	5.32%	3.93%	20.54%	2.43%	3.81%	3.58%
	0.87%	1.31%	0.32%	2.45%	3.89%	1.44%	2.62%	3.26%	1.09%	18.82%	3.29%	4.59%
	1.68%	1.46%	0.00%	1.18%	1.53%	0.96%	0.93%	2.33%	1.37%	5.43%	2.57%	6.28%
	0.43%	2.98%	0.06%	4.67%	5.64%	2.46%	0.13%	5.40%	3.12%	2.69%	2.14%	2.66%
	0.24%	1.05%	3.20%	3.22%	4.94%	5.67%	3.03%	1.11%	9.16%	2.38%	2.84%	11.76%
	0.48%	0.95%	0.00%	1.70%	3.30%	4.14%	4.46%	3.06%	1.38%	3.71%	5.46%	5.64%

Items	0d	7d	14d	21d	28d	35d	42d	49d	53d	55d	57d	64d
Bacteroides	2.36%	7.93%	10.54%	5.06%	3.02%	1.84%	0.82%	1.12%	0.48%	2.09%	2.40%	1.85%
	0.55%	12.64%	45.04%	6.37%	1.75%	0.24%	1.81%	1.23%	13.33%	2.72%	2.57%	0.64%
	0.70%	6.21%	11.28%	12.07%	3.92%	0.21%	1.79%	0.69%	0.96%	3.62%	2.05%	1.36%
	0.85%	19.12%	15.53%	7.97%	7.32%	1.78%	0.38%	1.33%	2.18%	1.20%	3.81%	4.47%
	3.95%	0.07%	4.21%	0.92%	0.61%	2.81%	3.13%	0.84%	2.33%	1.65%	2.45%	3.29%
	3.48%	1.26%	2.39%	2.52%	2.68%	4.85%	2.45%	3.38%	2.70%	1.42%	4.03%	3.16%
	0.62%	1.27%	6.28%	2.46%	1.81%	1.62%	2.24%	2.25%	1.95%	3.42%	1.65%	7.11%
	0.71%	2.60%	4.31%	0.74%	5.89%	5.80%	1.86%	1.27%	2.60%	2.17%	2.28%	1.07%
	0.56%	2.07%	0.62%	5.59%	4.03%	1.06%	1.85%	1.39%	1.42%	1.27%	1.23%	1.00%
	7..34%	7.03%	13.92%	4.59%	6.38%	2.00%	1.77%	3.06%	1.97%	1.96%	1.70%	3.54%
	1.82%	26.84%	5.20%	1.59%	1.76%	5.18%	2.73%	2.29%	2.36%	3.47%	1.39%	0.48%
	3.61%	49.30%	5.82%	2.18%	1.58%	1.49%	0.44%	0.97%	2.18%	9.69%	0.54%	1.30%
	14.43%	26.71%	22.16%	3.14%	1.05%	3.56%	2.03%	1.41%	2.42%	0.55%	2.09%	0.67%
	0.26%	3.33%	16.18%	12.27%	1.56%	2.81%	1.18%	3.64%	1.21%	1.39%	2.38%	0.19%
	1.73%	10.17%	7.93%	1.90%	0.88%	6.43%	0.10%	3.12%	0.89%	0.20%	3.38%	2.25%
	0.52%	17.23%	2.85%	2.31%	4.46%	2.08%	1.84%	1.69%	1.13%	1.07%	0.25%	0.46%
	1.29%	35.04%	1.72%	5.46%	0.78%	2.51%	4.31%	2.88%	0.93%	3.27%	0.59%	0.24%
	0.10%	33.17%	1.49%	9.95%	1.39%	2.51%	7.28%	0.69%	0.56%	0.85%	3.61%	0.49%
	1.38%	1.69%	17.55%	5.21%	1.90%	2.30%	0.85%	0.42%	0.96%	1.06%	3.99%	1.78%
	1.15%	1.61%	1.52%	2.29%	0.80%	0.54%	1.40%	1.08%	0.69%	4.61%	1.57%	1.49%
